# Long-term prognostic impact of left ventricular remodeling after a first myocardial infarction in modern clinical practice

**DOI:** 10.1371/journal.pone.0188884

**Published:** 2017-11-27

**Authors:** Christophe Bauters, Emilie Dubois, Sina Porouchani, Eric Saloux, Marie Fertin, Pascal de Groote, Nicolas Lamblin, Florence Pinet

**Affiliations:** 1 University of Lille, Inserm U1167, Institut Pasteur, University Hospital of Lille, Lille, France; 2 University of Lille, Inserm U1167, Institut Pasteur, Lille, France; 3 University Hospital of Lille, Lille, France; 4 University of Caen, EA 4650, University Hospital of Caen, Caen, France; I2MC INSERM UMR U1048, FRANCE

## Abstract

**Background:**

The association of left ventricular remodeling (LVR) after myocardial infarction (MI) with the subsequent risk of heart failure (HF) and death has not been studied in patients receiving optimal secondary prevention.

**Methods and results:**

We performed a long-term clinical follow-up of patients included in 2 prospective multicentric studies on LVR after first anterior MI. At 1-year echocardiography, LVR (≥20% increase in end-diastolic volume from baseline to 1 year) occurred in 67/215 (31%) patients in cohort 1 and in 87/226 (38%) patients in cohort 2. The prescription rate of secondary prevention medications was very high (ß-blockers at 1 year: 90% and 95% for cohorts 1 and 2, respectively; angiotensin-converting enzyme inhibitors or angiotensin II receptor blockers (ACE-I/ARB) at 1 year: 93% and 97% for cohorts 1 and 2, respectively). Median clinical follow-up after LVR assessment was 11.0 years in cohort 1 and 7.8 years in cohort 2. In both cohorts, LVR patients had a progressive increase in the risk of cardiovascular death or hospitalization for HF (p = 0.0007 in cohort 1 and 0.009 in cohort 2) with unadjusted hazard ratios of 2.52 [1.45–4.36] and 2.52 [1.23–5.17], respectively. Similar results were obtained when cardiovascular death was considered as an isolated endpoint. After adjustement on baseline characteristics including ejection fraction, the association with the composite endpoint was unchanged.

**Conclusion:**

In a context of a modern therapeutic management with a large prescription of evidence-based medications, LVR remains independently associated with HF and cardiovascular death at long-term follow-up after MI.

## Introduction

Myocardial infarction (MI) is a common complication of coronary artery disease with important prognostic implications [1, 2]. Left ventricular remodeling (LVR) after MI is a progressive dilation of the left ventricle that occurs in response to myocardial damage [3]. Studies performed before the modern era of MI management have identified LVR as a powerful indicator of a high risk of heart failure (HF) or cardiovascular death after MI [4, 5]. Post-MI LVR is commonly used as a surrogate endpoint in clinical studies [6–9]. However, the prognostic value of LVR has not been evaluated at long-term after MI in patients receiving reperfusion therapies and with systematic use of evidence-based medications. The aim of the present study was to report > 10 years of clinical follow-up of post-MI patients according to the absence/presence of LVR after MI. For this purpose, we analyzed the long-term cardiovascular outcome of patients included in two prospective multicentric studies on LVR after MI [10, 11].

## Methods

The REVE (REmodelage VEntriculaire) studies have been previously reported [10, 11]. REVE (i.e., cohort 1; inclusion period, February 2002 –June 2004; n = 266 patients) was designed to test the hypothesis that genetic polymorphisms in candidate genes may be associated with LVR [12]. REVE-2 (i.e., cohort 2; inclusion period, February 2006 –September 2008; n = 246 patients) was designed to analyze the association of circulating biomarkers with LVR [11]. Both studies were prospective with a multicentric recruitment. The inclusion criteria were the same: a first anterior Q-wave MI with ≥3 akinetic LV segments at predischarge echocardiography. Exclusion criteria were inadequate echographic image quality, life-limiting noncardiac disease, significant valvular disease, or previous Q-wave MI in both studies. In addition, patients >85 years and patients who had a scheduled coronary bypass graft were also excluded from cohort 1. The protocols were approved by the ethics committee of the Centre Hospitalier et Universitaire de Lille, Lille, France, and written informed consent was obtained from each patient. In both studies, the protocol required serial echocardiographic studies at baseline, 3 months, and 1 year after MI.

Echographic data were obtained by experienced ultrasonographers using commercially available second harmonic imaging systems. A standard imaging protocol was used based on apical 4- and 2-chamber views. All echocardiograms were recorded on optical disks and analyzed at the Lille Core Echo Laboratory as previously described [10, 11]. In both cohorts, LV volumes and ejection fraction (EF) were calculated using a modified Simpson’s rule. Intraobserver and interobserver variability in the evaluation of left ventricular end-diastolic volume (LVEDV) and left ventricular end-systolic volume (LVESV) has been previously reported [10]. Left atrial volume was measured as previously described [13]. In cohort 2, the level of B-type natriuretic peptide (BNP) and the E/Ea ratio were measured as previously reported [11, 14].

**Fig 1:**
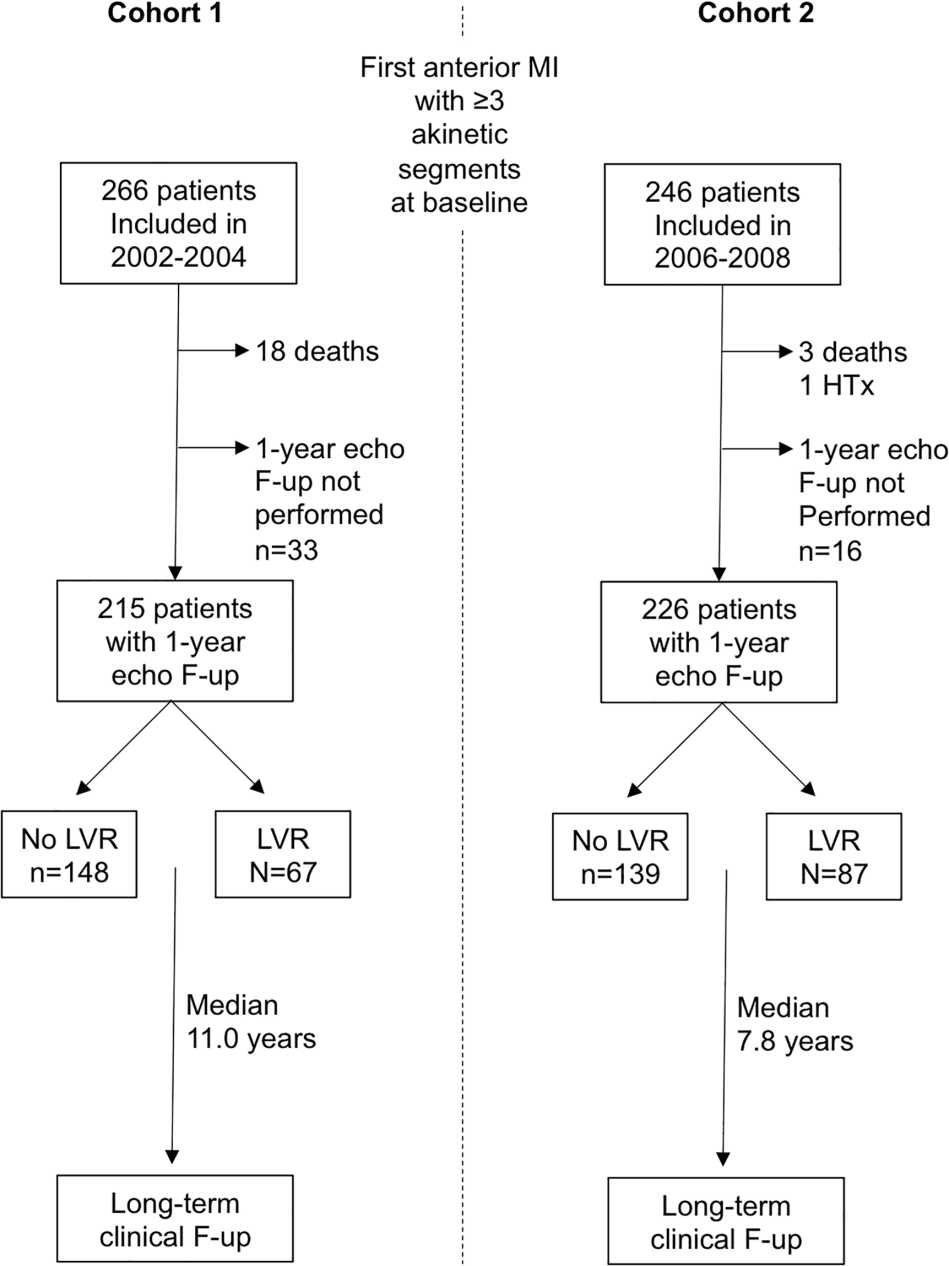
Flow chart of the study population. MI, myocardial infaction; HTx, heart transplantation; echo F-up, echocardiographic follow-up; LVR, left ventricular remodeling defined as ≥20% change in left ventricular end-diastolic volume from baseline to 1-year.

For the present analysis, we focused on the patients who underwent the 1-year echocardiographic follow-up. A flow chart of the study is shown in Fig 1. Overall, there were 512 included patients; 21 patients (with a mean LVEF of 37±8%) died during the first year of follow-up; 28 patients were hospitalized for HF during the first year of follow-up. In total, 441 patients (215 in cohort 1 and 226 in cohort 2) had an echographic study for evaluation of LVR. A long-term clinical follow-up on clinical events occurring after LVR assessment was performed by contacting the general practitioner or cardiologist, or the patients themselves. We collected data on death, hospitalization for heart failure, and recurrent MI. All events occurring during follow-up were adjudicated by two investigators with a third opinion in cases of disagreement. For hospitalizations during the follow-up period, hospital records were reviewed for evidence of clinical events. The events reported by the patients were systematically confirmed from the medical records. The primary endpoint of the study was cardiovascular death or hospitalization for heart failure. In both cohorts, the long-term outcome of patients with LVR was compared with that of patients with no LVR.

The change in LVEDV from the baseline echocardiogram to the 1 year echocardiogram was used as the indicator of LVR. In both cohorts, we used the cutoff value of 20% change in LVEDV to categorize the patients in the no LVR group (<20% change) or in the LVR group (≥20% change).

Information on cardiovascular treatments (antiplatelet therapy, ß-blockers, angiotensin-converting enzyme inhibitors (ACE-I), angiotensin II receptor blockers (ARB), aldosterone antagonists, and statins) was obtained at discharge from initial hospitalization, 3 months, 1 year, and 3 years post-MI. In addition, the doses of ß-blockers and ACEI/ARB were also recorded at discharge, at 3 months, and at 1 year. For each drug, the target dose was defined as the dose used in randomized trials with clinical endpoint reduction: acebutolol, 400mg/day; atenolol, 100mg/day; bisoprolol, 10mg/day; carvedilol, 50mg/day; metoprolol, 200mg/day; nebivolol, 10mg/day; captopril, 150mg/day; enalapril, 20mg/day; fosinopril, 20mg/day; lisinopril, 20mg/day; perindopril, 8mg/day; ramipril, 10mg/day; trandolapril, 4mg/day; candesartan, 32mg/day; losartan, 100mg/day; valsartan, 320mg/day.

The cause of death was determined after a detailed review of the circumstances of death and classified as cardiovascular or non-cardiovascular. Deaths from unknown cause were considered as cardiovascular. Hospitalization for HF was defined as hospitalization for symptoms of dyspnea or edema associated with bilateral rales, elevated venous pressure, or interstitial or alveolar edema on chest X-ray, or the addition of intravenous diuretics or inotropic medications. Myocardial infarction was defined according to the universal definition [15].

Continuous variables were described as the mean ± standard deviation (SD) and were compared by the paired Student’s t-test or the unpaired Student’s t-test as appropriate. Categorical variables were presented as absolute numbers and percentages and were compared using chi2 analysis or Fisher’s exact test as appropriate. Cumulative rates of the composite endpoint (cardiovascular death or hospitalization for HF) and of cardiovascular death were estimated using the Kaplan-Meier method and difference between the no LVR group and the LVR group were tested with the Log-rank test. Hazard ratios and 95% confidence intervals (CI) were calculated by the Cox model. For these analyses, LVR was used as a categorical variable (<20%, ≥20%). Adjusted analyses included age, sex, diabetes mellitus, baseline LVEF, and baseline LVEDV. The proportional hazards assumption was tested for each variable. All statistical analyses were performed with the STATA 14.1 software^®^ (STATA Corporation, College Station, Texas, USA). Statistical significance was assumed at p-value <0.05.

## Results

One-year echocardiographic follow-up was performed in 215 of 266 included patients in the REVE study (cohort 1) and in 226 of 246 included patients in the REVE-2 study (cohort 2). Table 1 shows the baseline characteristic (at time of initial hospitalization for MI management) of the patients. The patients in cohort 2 were more often treated by primary PCI and less often by thrombolysis; otherwise, the baseline characteristics were similar. The mean LVEF was 50% in both cohorts; the proportion of patients with a LVEF <40% was 13% in cohort 1 and 12% in cohort 2. The rate of patients with Killip class 2 or higher was 22% in cohort 1 and 31% in cohort 2.

**Table 1:** Baseline characteristics of the patients who completed the 1-year echocardiographic follow-up in both cohorts.

	Cohort 1 (Inclusion period 2002–2004)			Cohort 2 (Inclusion period 2006–2008)		
	All patients (n = 215)	No LVR (n = 148)	LVR (n = 67)	All patients (n = 226)	No LVR (n = 139)	LVR (n = 87)
Age, years	58±13	58±13	59±13	57±14	57±14	57±13
Women, %	26	22	36*	19	17	23
History of hypertension, %	46	41	55	37	42	29*
Diabetes mellitus, %	20	18	25	19	20	18
Reperfusion therapy, %						
- Primary PCI	27	26	29	52	51	54
- Thrombolysis	58	56	61	36	34	38
- None	15	18	10	12	15	8
Multivessel CAD, %	35	34	39	41	39	45
PCI during hospitalization, %	91	90	93	88	87	88
Stent implantation in patients with PCI, %	98	98	98	98	98	97
Final TIMI grade 3 flow in infarct-related vessel, %	86	88	82	88	86	91
LV ejection fraction, %	50±9	51±9	47±9†	50±8	51±8	47±8†

Data are mean±SD or percentages.

LVR, left ventricular remodeling (≥20% increase in end-diastolic volume from baseline to 1 year); PCI, percutaneous coronary intervention; CAD, coronary artery disease; TIMI, Thrombolysis In Myocardial Infarction; LV, left ventricular.

* p<0.05 vs No LVR;

^†^ p<0.005 vs No LVR.

As previously reported [10, 11], there was a significant increase in LVEDV from 56.4±14.7 ml/m^2^ at baseline to 62.8±18.7 ml/m^2^ at 1 year in cohort 1, p<0.0001; and from 52.3±13.8 ml/m^2^ at baseline to 62.3±18.4 ml/m^2^ at 1 year in cohort 2, p<0.0001. When defined as a ≥20% increase in LVEDV from baseline to 1 year, LVR was observed in 67 patients (31% of population with echocardiographic follow-up) in cohort 1, and in 87 patients (38% of population with echocardiographic follow-up) in cohort 2. When compared to patients without LVR, patients with LVR had a significantly lower LVEF at baseline in both cohorts (Table 1). Cardiovascular treatments at baseline (hospitalization for MI), 3 months, 1 year (evaluation of LVR), and 3 years are listed in Table 2. Overall, the prescription rates of secondary prevention medications were very high at each time point. Except for aldosterone antagonists which were more often used in patients with LVR, the prescription rates did not differ according to LVR. Beta-blockers and ACE-I/ARB were used in 85 to 100% of the cases in both cohorts. S1 Table shows the percent of target doses achieved for ß-blockers and ACE-I/ARB throughout the first year of follow-up. The doses were in the 60–70% range for both types of drugs.

**Table 2:** Cardiovascular treatments at baseline, 3 months, 1 year, and 3 years.

	Cohort 1			Cohort 2		
	All patients (n = 215)	No LVR (n = 148)	LVR (n = 67)	All patients (n = 226)	No LVR (n = 139)	LVR (n = 87)
Antiplatelet therapy,%						
- Baseline	99	100	99	100	100	100
- 3 months	97	97	97	100	100	100
- 1 year	95	98	90*	99	100	99
- 3 years	93	93	91	96	95	98
ß-blockers, %						
- Baseline	94	94	96	97	98	97
- 3 months	92	93	91	96	96	96
- 1 year	90	91	90	95	94	95
- 3 years	86	87	84	88	88	89
ACE-I or ARB, %						
- Baseline	98	97	99	98	99	95
- 3 months	95	95	94	99	98	100
- 1 year	93	91	96	97	96	98
- 3 years	89	85	97*	92	93	92
Aldosterone antagonists, %						
- Baseline	10	8	13	34	32	36
- 3 months	13	9	24†	34	33	35
- 1 year	12	7	22†	34	29	41*
- 3 years	10	7	16	29	23	39*
Statins, %						
- Baseline	99	99	97	94	94	95
- 3 months	94	95	93	97	96	99
- 1 year	93	93	91	95	95	95
- 3 years	88	90	85	90	90	90

Data are percentages.

LVR, left ventricular remodeling (≥20% increase in end-diastolic volume from baseline to 1 year); ACE-I, angiotensin-converting enzyme inhibitors; ARB, angiotensin II receptor blockers.

* p<0.05 vs No LVR;

^†^ p<0.005 vs No LVR.

**Fig 2:**
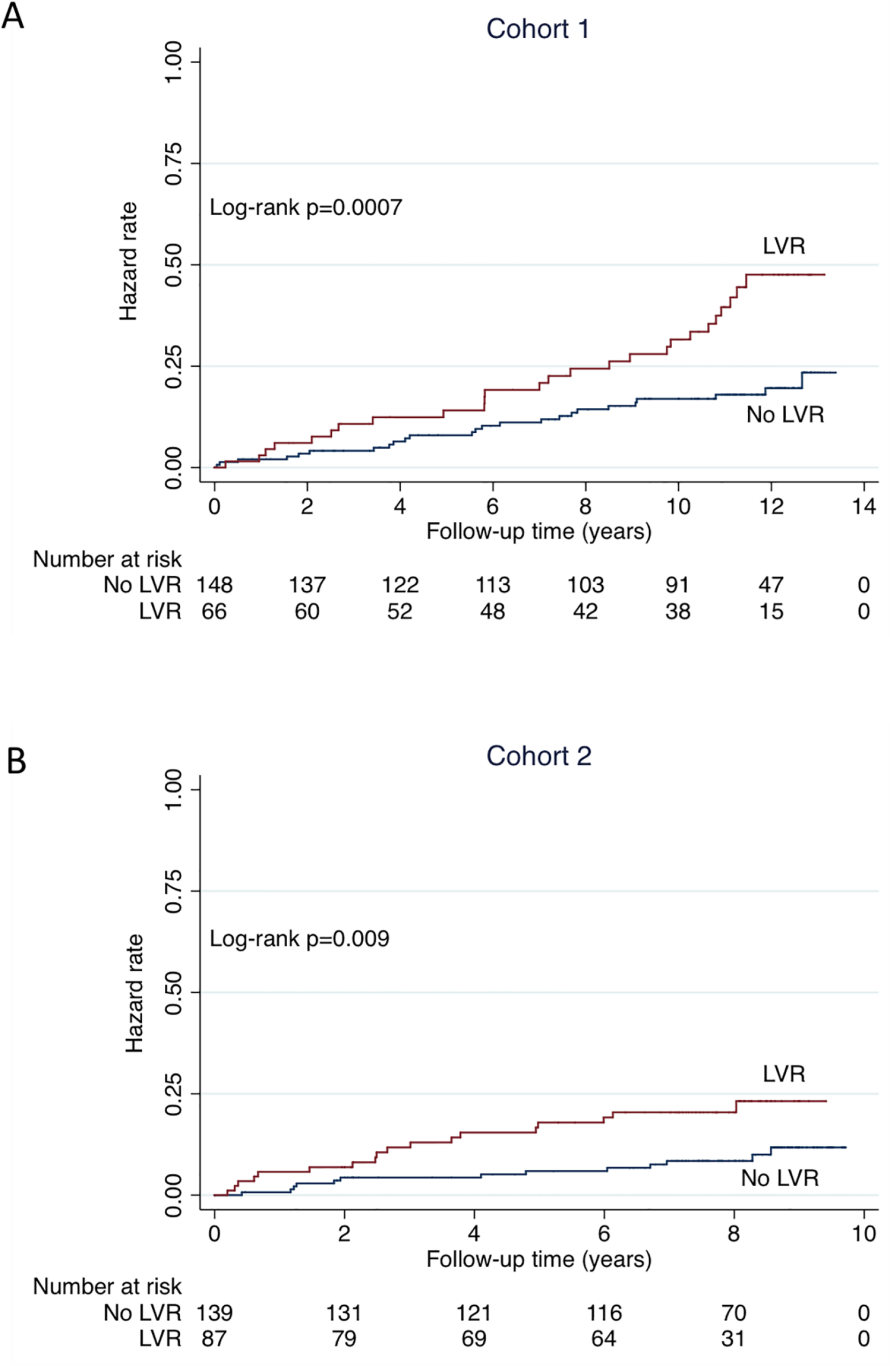
Unadjusted Kaplan-Meier curves for the composite endpoint of cardiovascular death or hospitalization for HF. The study population is divided in 2 groups according to LVR at 1-year follow-up (≥20% change in left ventricular end-diastolic volume from baseline to 1 year). Follow-up is starting at the time of 1-year echocardiography. A = cohort 1; B = cohort 2.

**Fig 3:**
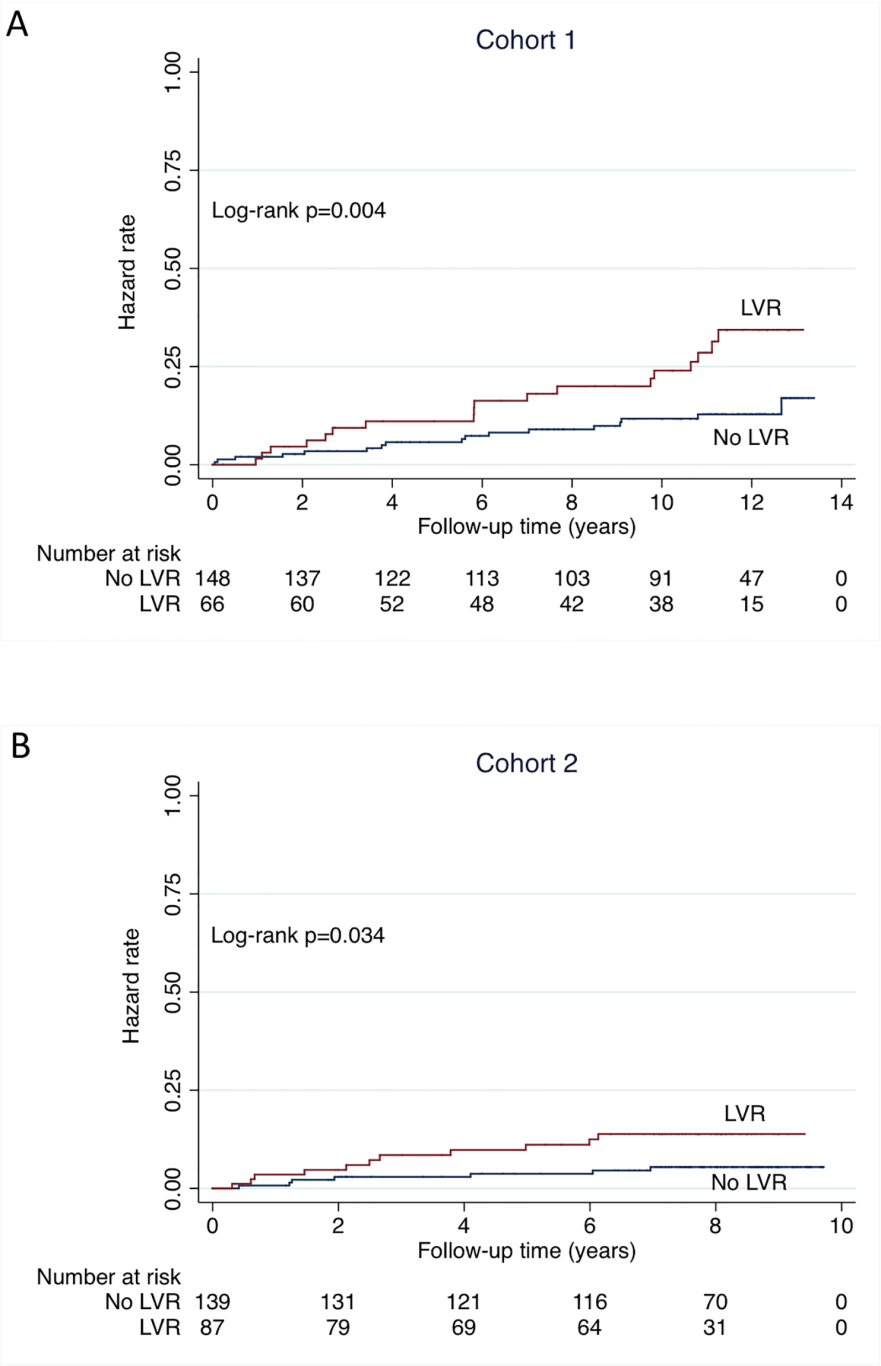
Unadjusted Kaplan-Meier curves for cardiovascular death. The study population is divided in 2 groups according to LVR at 1-year follow-up (≥20% change in left ventricular end-diastolic volume from baseline to 1 year). Follow-up is starting at the time of 1-year echocardiography. A = cohort 1; B = cohort 2.

Clinical follow-up data were obtained for 214 patients at a median of 11.0 years after the 1-year echocardiographic follow-up in cohort 1, and for 226 patients at a median of 7.8 years after the 1-year echocardiographic follow-up in cohort 2. Thirty-four patients were hospitalized for HF and 70 patients died (35 from cardiovascular causes) in cohort 1. Twenty-one patients were hospitalized for HF and 31 patients died (18 from cardiovascular causes) in cohort 2. The composite endpoint of cardiovascular death or hospitalization for HF occurred in 51 patients in cohort 1 and in 31 patients in cohort 2. Fig 2 shows Kaplan-Meier curves for the composite endpoint over the follow-up period. In both cohorts, LVR patients had a progressive increase in risk compared with no LVR patients. The unadjusted HRs (LVR vs no LVR) for the composite endpoint were 2.52 [1.45–4.36], p = 0.001, and 2.52 [1.23–5.17], p = 0.012, in cohort 1 and cohort 2, respectively. As shown in Fig 3, the patients with LVR also had an higher risk of cardiovascular death when considered as an isolated endpoint. After adjustment for baseline characteristics (age, sex, diabetes mellitus, baseline LVEF, and baseline LVEDV), the association of LVR with the composite endpoint was unchanged (HRs = 2.20 [1.20–4.04], p = 0.011, and 2.57 [1.18–5.56], p = 0.017, in cohort 1 and cohort 2, respectively). During the follow-up period, there were 26 patients with recurrent MI in cohort 1 and 18 in cohort 2. In both cohorts, the proportion of patients with recurrent MI was similar in the no LVR group and in the LVR group (not shown).

Finally, we analyzed the associations of different echographic and biological parameters, assessed at 1 year after MI, with cardiovascular death or hospitalization for heart failure at long-term follow-up (Table 3). In both cohorts, LVEDV, LVESV, LVEF and atrial volume were all strongly associated with the clinical outcome; in addition, E/Ea ratio and BNP were also strongly associated with outcome in cohort 2 (parameters not available in cohort 1). Multivariate analysis was not performed due to important collinearity between variables.

**Table 3:** Echographic and biological parameters assessed at 1 year after MI as predictors of cardiovascular death or hospitalization for heart failure at long-term follow-up.

	Cohort 1			Cohort 2		
	HR [95% CI]	Wald Statistic	P	HR [95% CI]	Wald Statistic	P
LVEDV (ml/m2)	1.03 [1.02–1.04]	4.36	<0.0001	1.02 [1.01–1.04]	3.28	0.001
LVESV (ml/m2)	1.04 [1.02–1.05]	5.06	<0.0001	1.04 [1.02–1.05]	5.15	<0.0001
LVEF (%)	0.94 [0.92–0.96]	-4.65	<0.0001	0.91 [0.88–0.93]	-6.27	<0.0001
LAV (ml/m2)	1.04 [1.01–1.06]	3.10	0.002	1.08 [1.03–1.13]	3.76	<0.0001
E/Ea	NA			1.13 [1.06–1.21]	3.60	<0.0001
BNP (log[pg/ml])	NA			2.76 [2.00–3.80]	6.20	<0.0001

HR indicates hazard ratio by univariate Cox regression; CI, confidence interval; LVEDV, left ventricular end-diastolic volume; LVESV, left ventricular end-systolic volume; LVEF, left ventricular ejection fraction; LAV, left atrial volume; E/Ea, early rapid filling wave/mitral annular early diastolic velocity; BNP, B-type natriuretic peptide; NA, not available.

## Discussion

Left ventricular remodeling is nowadays considered as a surrogate for adverse events (HF, cardiovascular death) after MI [16]. Since LVR is easy to measure using non-invasive imaging techniques, it is extensively used as endpoint in studies testing new therapeutic interventions [6–8] or searching for prognostic biomarkers [9, 11, 12, 17–20] in MI patients. In the echocardiographic substudy of the Survival and Ventricular Enlargement (SAVE) trial, progressive LV dilation after MI was associated with a higher risk of both HF and cardiovascular death [4]. Inclusions in this clinical trial however took place nearly 3-decades ago, and since then there has been major improvements in MI management with an impact on outcome, including systematic use of reperfusion therapies and secondary prevention medications. Angiotensin-converting enzyme inhibitors or ARB, ß-blockers, and—for the higher risk patients—aldosterone antagonists are now standard therapy in the post-MI setting since their use has been associated with major improvements in prognosis [21–24]. Whether the combination of these therapeutics may have altered the relationship between LVR and subsequent outcome is currently unknown. Although analyses from other cohorts [5, 25] have replicated the results of the SAVE substudy, this was not in a context of systematic use of reperfusion, ß-blockers, and ACE-I/ARB. More recently, in an analysis of the Valsartan in Acute Myocardial Infarction (VALIANT) echo study database [26], Solomon et al. reported that baseline echocardiographic measurements were extremely predictive of cardiovascular outcomes but could not assess the influence of changes in ventricular size on subsequent outcomes because of insufficient follow-up time after the final echocardiogram.

To the best of our knowledge, the present study is the first to present more than 10 years of follow-up after a prospective evaluation of LVR in patients with high use of reperfusion therapies, nearly systematic use of ACE-I/ARB and ß-blockers, and antialdosterone use in selected patients. The doses of ACE-I/ARB and ß-blockers were relatively high for a real-life study, reaching 60–70% of the target dose at time of LVR assessment. It should also be pointed out that coronary stents were used in most patients and that a normal flow in the infarct-related artery was achieved in the great majority of the cases. In spite of this medical management, LVR was associated with a higher risk of HF and cardiovascular death with very consistent results in the two independent cohorts of patients. The HR of 2.5 for a ≥20% increase in LVEDV from baseline to 1 year illustrates the magnitude of the effect. Importantly, when assessed at 1 year after MI, LVR provides independent prognostic information on top of echographic variables (LVEF, LVEDV) measured at baseline. Finally, although some events occurred early, the curves continued to diverge late and very late after MI as shown for cohort 1 (Figs 2A and 3A). This suggests that, in the modern era of MI management, LVR is not associated with major immediate consequences but is more probably a process with lifelong prognostic implications.

Several study limitations should be discussed. Firstly, our results were obtained in relatively young patients with first anterior MI and substantial residual akinesia at predischarge echocardiography; they may not apply to all post-MI patients. On one hand, our patients are at higher risk than many patients who are nowadays discharged after MI with no or minimal residual akinesia. On the other hand, our patients, with a mean LVEF of 50%, are at lower risk than patients selected on the basis of heart failure and/or left ventricular systolic dysfunction during initial hospitalization. As an example, the combined cardiovascular endpoint of cardiovascular death, reinfarction or hospitalization for heart failure was reached by more than 30% of the patients included in the VALIANT trial after only 36 months of follow-up [22]–a rate much higher than in our study. Another indicator of the moderate cardiovascular risk of our patient population is the relatively high proportion of deaths classified as non-cardiovascular (50% in cohort 1; 42% in cohort 2); this proportion is higher than what is expected from a HF population [27] but lower than what has been reported in patients with stable coronary artery disease [28]. Secondly, although most patients had acute reperfusion, the proportion of patients with primary PCI reflects the practice in 2002–2004 and 2006–2008 and was lower that it would be nowadays. This type of limitation is inherent in all studies with long-term follow-up. Thirdly, it should be underscored that there is no uniform definition of LVR after MI. The definition of a ≥20% increase in LVEDV from baseline to 1 year was prospectively chosen when the REVE and REVE-2 studies were designed [10, 11]. Our results (Table 3) show that LVESV is also a good indicator of long-term outcome. Finally, we have no information on cardiovascular treatments after 3 years and there might have been a decrease in prescription rates during the subsequent years. Major changes are however unlikely as suggested by the results of a study from the same geographical area in 2010–2011, recruiting patients with stable coronary artery disease (median time since last MI = 5 years) in whom ß-blockers and ACE-I/ARB were prescribed in 79% and 82% of the patients, respectively [29].

The implications of our results are threefold. Firstly, in the modern era of MI and HF management, LVR remains a strong surrogate for adverse long-term outcome and can still be used as endpoint in clinical studies. Secondly, after a large MI, systematic echocardiographic follow-up for quantifying LVR should be recommended as it provides important prognostic information on top of baseline variables. Thirdly, our data suggest that further effort is needed to reduce LVR and its consequences in post-MI patients. There is ongoing research in this area. Experimental studies have shown a potential impact of the new angiotensin receptor-neprilysin inhibitor LCZ696 on LVR [30] and a large clinical trial with this drug is in progress in patients after MI (clinicalTrials.gov Identifier: NCT02924727).

In summary, in spite of a therapeutic management comprising ß-blockers, ACE-I/ARB, and aldosterone antagonists, LVR was independently associated with HF and cardiovascular death at long-term follow-up after MI.

## Supporting information

S1 Table Percent of target doses achieved throughout first year follow-up for patients receiving secondary medical prevention. (DOCX)
