# Supplementary material for: Long-term prognostic impact of left ventricular remodeling after a first myocardial infarction in modern clinical practice
Source: PLoS One. 2017 Nov 27;12(11):e0188884. doi: 10.1371/journal.pone.0188884 (PMC5703528; doi:10.1371/journal.pone.0188884)
Supplement: S1 Table — (DOCX) [file pone.0188884.s001.docx]

**S1 Table. Percent of target doses achieved throughout first year follow-up for patients receiving secondary medical prevention.**

|  | **Baseline** | **3 months** | **1 year** |
| --- | --- | --- | --- |
| ß-blockers |  |  |  |
| - Cohort 1 | 62±31 (n=203) | 61±32 (n=198) | 63±32 (n=194) |
| - Cohort 2 | 55±29 (n=220) | 65±30* (n=214) | 70±30*† (n=214) |
|  |  |  |  |
| ACE-I/ARB |  |  |  |
| - Cohort 1 | 62±33 (n=210) | 64±32 (n=204) | 63±31 (n=199) |
| - Cohort 2 | 60±30 (n=221) | 69±31* (n=220) | 73±30* † (n=219) |

Data are mean±SD of target doses of medications as defined in methods section.

ACE-I, angiotensin-converting enzyme inhibitors; ARB, angiotensin II receptor blockers.

* p<0.0001 vs Baseline; † p<0.05 vs 3 months.
